# Comparing Prices for Food and Diet Research: The Metric Matters

**DOI:** 10.1080/19320248.2015.1095144

**Published:** 2016-04-25

**Authors:** N. R. V. Jones, P. Monsivais

**Affiliations:** ^a^ Centre for Diet and Activity Research, MRC Epidemiology Unit, University of Cambridge School of Clinical Medicine, Institute of Metabolic Science, Cambridge Biomedical Campus, Cambridge, UK

**Keywords:** Food prices, measurement, research methods, economics, food security, access

## Abstract

An important issue in research into access to healthy food is how best to compare the price of foods. The appropriate metric for comparison has been debated at length, with proponents variously stating that food prices should be compared in terms of their energy content, their edible mass, or their typical portion size. In this article we assessed the impact of using different food price metrics on the observed difference in price between food groups and categories of healthiness, using United Kingdom consumer price index data for 148 foods and beverages in 2012. We found that the choice of metric had a marked effect on the findings and conclude that this must be decided in advance to suit the reason for comparing food prices.

## Introduction

An essential component of food access and food security is the notion that consumers have sufficient resources for a nutritious diet.^1^ Thus, it is important to standardize methods for comparing the cost of different foods and beverages (hereafter collectively referred to as *foods*) to examine how prices may affect access to a nutritionally adequate diet. Having appropriate metrics for comparison is also important for developing public health policies that recommend substituting some foods for others, if they are to suitably account for the potential limiting factor of cost.

Though food prices are currently monitored by governments, international agencies, and private organizations, such tracking is usually not suitable for comparing the costs of different foods in relation to their nutritional value.^2^ This has implications for monitoring food security, given that the relationship between nutritional value and prices is a factor that will have a large impact on food security. An inadequate understanding of how the prices of different foods compare could also limit the effectiveness of food assistance and nutrition programmes, such as the Supplemental Nutrition Assistance Program in the United States or the UK’s Healthy Start, because these programs are predicated on the assistance being large enough to purchase foods that contribute to a healthy diet. As such, there is arguably a requirement to be able to fairly compare food prices, but there is an ongoing and vigorous debate as to the best way to make such comparisons, with different researchers favoring different metrics and finding alternatives to be misleading.^3^ ^,4^ Though this is ostensibly an economic issue, the purpose of exploring it here is to improve the methodology available for research into the economic determinants of food insecurity, hunger, and malnutrition.

In this article we consider earlier arguments for the different metrics of food price comparison and then apply these metrics to national food price data from the UK, exploring how the choice of metric can influence the findings of research on the question of whether healthier foods are more expensive. We then make suggestions as to how future research should express food prices to ensure that reported results are meaningful answers to the question being asked, providing reasons for and against using different metrics and examples of each.

### The debate so far

The need for a common metric by which foods can be assessed is due to the different unit sizes of purchased goods, meaning that the price needs to be divided by some quality of the food in question so that it is comparable to others. For example, if we want to compare the price of an orange with the price of a bag of apples there are numerous ways to do this: we could compare the individual orange to the bag or in terms of mass, energy, typical portion size, or perhaps other quantities of the foods. Comparisons have typically been made by comparing the price divided by the energy content or mass of the food in question.^5^ However, both of these approaches have received criticism^3^ ^,4^ and neither has been adopted unanimously by researchers in this field.

One of the earliest published efforts to consider the price of food in terms of its content was made by Atwater in 1894,^6,^ ^7^ who recognized that the nutritive value of foods differed and that their cost ought to be framed in terms of the energy or nutrients they provide. The price per unit of energy metric has become a widely used approach for researchers interested in food prices and nutrition, with a range of different authors using it in different study designs and in different countries.^8^ ^–^ ^10^ Moreover, the costing of foods in terms of price per unit energy in development economics suggests the utility of this metric for understanding how food prices affect diet in low-income populations.^4^ ^,11,^ ^12^

However, assessing price in terms of energy has been criticized on a number of grounds, namely, that people do not purchase foods in forms that are easily comparable in terms of calories, meaning that the price per calorie may not be relevant to consumer behavior.^13^ Similarly, foods are not necessarily eaten in isoenergetic quantities, so comparisons between foods typically eaten in quantities that do not provide equal amounts of energy—for example, carrots and ice cream—is meaningless in practical terms.^14^ Finally, there has been criticism of attempting to assess the relationship between how healthy a food is and its price when price is measured in terms of energy and the healthiness of a food is measured by its energy density, because this can lead to autocorrelation.^3^ ^,5^ The critics of an energy-based metric instead often propose the use of mass as the most appropriate metric.^5^ Proponents on both sides of the debate have engaged in studies using randomly generated data to investigate the apparent mathematical flaws in the other’s choice of metric, with opposing results.^7^ ^,15^

A third alternative, the use of portion size as a price metric, has also been proposed on the grounds that comparisons of prices based on a fixed amount of calories or mass of food may have little behavioral relevance. Portion sizes are a better reflection of the quantities of foods that are typically consumed and these can vary substantially among different types of foods.^5^ This method would allow for a more realistic comparison of different foods and for the cost figures to be more readily appraised. However, this metric requires up-to-date and accurate data concerning the quantity typically consumed, which may not always be available or appropriate for all populations, making it harder to adopt as a standard.

In summary, this debate is sharply polarized and does not appear to be reaching a resolution. Accordingly, it is important to find common ground and identify instances where there is agreement about what most appropriate metric is the given the question being asked.

### Empirical analysis

We analyzed government food price data from the UK to explore how the choice of food price metric can have on the results produced by research exploring which foods are more expensive.

## Methods

### Food price data

We obtained national food price data used to calculate the UK Consumer Price Index (CPI) from the Office for National Statistics and matched them to a range of appropriate items in the National Diet and Nutrition Survey (NDNS),^16^ using a method described previously.^17^ Briefly, this was done by matching each CPI item to a range of NDNS foods deemed to be a good match. Following this, mean nutrient values were produced for each CPI item by calculating the mean of the nutrient values listed for the NDNS matches. To reflect the different ways in which a food may be prepared for consumption and the implications this would have for its nutrient content as listed in NDNS (which lists foods as consumed, after adjustments made in preparation), we weighted these mean values by the frequency with which each preparation method is recorded in NDNS, intending to accurately reflect the ways the foods are consumed by people in the UK. Prices were then adjusted for edible portion of each food using *United States Department of Agriculture’s Handbook 102* ^18^ to account for the fact that the price data were for foods as purchased and the NDNS data for foods as consumed. We assigned a portion size to each food item using portion sizes typically consumed in the UK, based on Wrieden and Barton.^19^ Items are included in the CPI if they are frequently consumed by many households because the index aims to sample goods and services that are typical of expenditure in the UK.^20^ As such, the foods included in it can reasonably be expected to represent those foods typically consumed.

### Classification of foods

We determined which foods were more and less healthy using the Food Standards Agency’s WXYfm nutrient profiling model (hereafter referred to as the FSA Score), which provides a categorical definition of a food’s healthiness based upon energy, saturated fat, total sugar, sodium, fiber, protein, and fruit, vegetable, and nut content.^21^ We assigned foods to food groups in line with the UK’s *Eatwell Plate*, a government-produced nutrition communication tool, using a table in *The Livewell Report*, which matched NDNS food categories to Eatwell food groups.^22^ These steps resulted in a data set of 148 foods and beverages with information on their food groups, whether they are more or less healthy, and their mean 2012 price per unit of energy, per unit of mass, and per portion.

### Statistical analysis

Tests for a significant difference in mean prices by group were conducted using a *t* test for comparing more and less healthy foods and analysis of variance for comparing the Eatwell food groups. All analyses were conducted using Stata (Ver SE 12.1).^23^ Figures were produced using *R* (version 3.0.2 for Windows) and the *ggplot2* package.^24^ ^,25^

## Results

Table 1 reports the results of our analyses, containing the mean and standard error for each price metric by FSA score–defined healthiness and by food group. Prices were calculated on March 15, 2015, at an exchange rate of U.S.$1.47 per £1.The overall mean price per 100 g was £0.57 ($0.84), £0.50 ($0.74) per 100 kilocalories, and £0.47 ($0.69) per portion, with similar standard errors (0.05 for mass- and energy-based prices, 0.04 for portion-based prices). The results by FSA category show the impact of the choice of denominator on the observed relationship between how healthy a food is and its price: healthier foods were significantly more expensive in terms of energy (0.65 £/100 kcal ($0.96) versus 0.28 £/100 kcal ($0.41), *P* < .001) and per portion (0.55 £/portion ($0.81) versus 0.35 £/portion ($0.52), *P* < .035) yet significantly less expensive in terms of mass (0.46 £/100g ($0.68) versus 0.85 £/100g ($1.25), *P* < .001). When prices were examined by Eatwell group, the choice of price metric also exerted an effect on the results, with fruit and vegetables being the least expensive food group by mass (0.28 £/100g ($0.41)) and second least by portion (0.25 £/portion ($0.37)) yet the most expensive in terms of calories (0.83 £/100 kcal ($1.22)). Meat and other sources of a protein were the most expensive group by mass (0.93 £/100 g ($1.37)) and portion size (0.94 £/portion ($1.39)), yet in the middle of the price range given their energy content (0.52 £/100 kcal ($0.77)). In summary, these results show that there is a considerable difference in the cost of different foods depending on the price metric chosen.

**Table 1.** Mean price, in UK Pounds Sterling^a^, per 100 g, per 100 kcal, and per portion for foods and beverages by FSA score category and Eatwell food group, using prices from the Consumer Price Index for the third quarter of 2012, *n* = 148^b^.

		Price per 100 g (£)		Price per 100 kcal (£)		Price per portion (£)	
	*n*	Mean	Standard error	Mean	Standard error	Mean	Standard error
Total	148	0.57	0.05	0.50	0.05	0.47	0.04
FSA score category							
More healthy	87	0.46	0.05	0.65	0.08	0.55	0.07
Less healthy	61	0.85	0.09	0.28	0.03	0.35	0.04
*P* value of 2-tailed *t* test		<.001		<.001		.035	
Eatwell food group							
Bread, rice, potatoes, pasta	18	0.32	0.08	0.11	0.02	0.18	0.04
Fruit and vegetables	38	0.28	0.03	0.83	0.15	0.25	0.04
Milk and dairy foods	14	0.70	0.16	0.44	0.11	0.39	0.14
Meat, fish, eggs, beans, other sources of protein	36	0.93	0.08	0.52	0.06	0.94	0.11
Food and drinks high in fat and/or sugar	42	0.77	0.13	0.36	0.10	0.40	0.08
*Pvalue of one-way analysis of variance*		*<.001*		*<.001*		*<.001*	

^a^$1.47 US per £ 1 UK on 15 March 2015.

^b^FSA indicates Food Standards Agency.

## Discussion

Our results show that the unit of comparison has an impact on which foods were the most expensive. The effect of this can be to completely change the relationship observed, which has considerable implications for any research on food costs or food security. This finding is in line with previous work,^5,^ ^14^ which also found that fruits and vegetables went from being the most expensive to the least expensive food group depending on the price metric. As has been previously stated,^5^ this finding is likely due to the low energy density of fruits and vegetables, which results in a low price per gram and a high price per calorie.

Our findings have important implications for research comparing the price of different foods, given the impact that choosing an inappropriate metric would have on the results. Rather than promote any one food price metric to the exclusion of others, here we will suggest guidance as to when it is most appropriate to use the different metrics, given the research question being asked. Such guidance would enable researchers to select price metrics most appropriate for their research and also make the comparison of different studies easier, thus allowing information to be better pooled and used to inform food policy.

### When to use mass as a metric

We now set out what we think are the circumstances in which each of the three price metrics assessed in this article are the most salient. When the research question makes a comparison between similar foods, the use of mass as the price comparison metric may be the most appropriate given that this allows the consumer to determine whether two products that will serve the same purpose within their diet differ in price. An example of this is the comparison between different packets of butter: both are nutritionally similar and will be served in similar portions but there may be a price difference between them. This metric seems most appropriate here because in some countries, including the UK^26^ and Australia,^27^ this unit price (price per unit weight) labeling is mandated and therefore is available to consumers when making decisions. However, the mass of the product bears little relationship to how it is consumed and the level of sustenance it provides, meaning that this metric is unlikely to be a meaningful way to compare very different types of food; for example, steak and lettuce. It should be noted that the price per unit of mass will not be available in all food outlets and in many countries and this should be taken into account by researchers deciding on a price comparison metric if they want it to have any behavioral relevance.

### When to use portions as a metric

Dietary guidelines often include recommendations for consuming a specific number of portions of food per day or per week. Given the observed relationship between the price of more and less healthy foods, concerns may exist that the cost of recommended foods might prevent some groups from meeting these guidelines.^17^ ^,28^ In such circumstances, the price-per-portion metric may be useful for estimating the likely impact on consumer costs, allowing guidelines to be adapted accordingly. For example, U.S. government analysis has estimated the cost of meeting the recommended 5 portions of fruit and vegetables per day.^29^

Alternatively, when researchers wish to determine the cost of directly substituting one food for another, the use of portion sizes is appropriate given that the foods may be of different quantities of both energy and mass. This metric allows for a comparison to be made that is directly connected to how people typically consume the foods being compared. For example, when comparing the cost of serving of shepherd’s pie and a grilled cod fillet, the portion size is probably the most appropriate way to compare these foods, which differ in energy and nutrient contents but have a similar role in a meal. Studies have used this approach to examine the cost of substituting healthier foods for less-healthy foods in institutional settings^28^ and the cost and nutritional effects of substituting fruit for fruit juice in the diets of children.^30^ This approach is also likely to lead to results that are more readily interpretable, in contrast to prices being expressed in terms of energy or mass, which has advantages when it comes to dissemination to a lay audience. However, the use of a typical portion size relies on these data being available, which will not always be the case.

### When to use energy as a metric

When addressing questions of public health and nutrition, pricing food on the basis of energy content appears to be the most appropriate approach because the comparison is between the sustenance the foods can contribute. This is based on the notion of physiological energy requirements for maintaining energy balance, with foods varying in the extent to which they contribute to achieving this energy target. In contrast, the mass and number of portions have no physiological basis. To give an example, this metric would be the best way to compare bread and apples, foods that are likely to be very different in terms how much energy they can provide when matched mass for mass or portion for portion. There are variations of this approach that can also be used, such as estimating the cost of obtaining a specified quantity of nutrients—for example, protein,^6^ potassium, or fiber^31^; however, energy is the key component of food required to sustain life in the short term, so for general questions concerning public health nutrition it will be suitable. However, as with portion sizes, the data required to calculate the price in terms of energy content will not always be available to researchers.

Table 2 summarizes our considerations when selecting a food price metric. We hope that our suggestions as to the most appropriate metric for use in different situations may bring about an increased awareness of the notion that one metric cannot be used to answer all questions. However, these considerations are likely to be contested and we welcome debate over the advice we set out here.

**Table 2.** Three commonly used food price metrics and indication of when their application is most appropriate.

Food price metric	Price per unit of mass	Price per portion	Price per unit of energy
Best used for	Comparing prices of nutritionally similar foods in the context of consumer choice, where mass is likely to be the only product information available to the purchaser.	Comparing prices in the context of direct substitutions of one food for another. This is likely to be of use for analyzing food-based policies that promote such substitutions.	Comparing prices in the context of public health and food security, where the quality of a diet consumed for survival is of concern.
Potential concerns	Limited relevance for assessing the cost of abating hunger or achieving sustenance.	Requires accurate and appropriate portion size data for the population being studied and portions estimated for a different population may lead to inaccurate findings being reported if misapplied.	Calorie information is not always available. Calorie-based comparisons might not be relevant for some consumer decision making.

**Figure 1.**
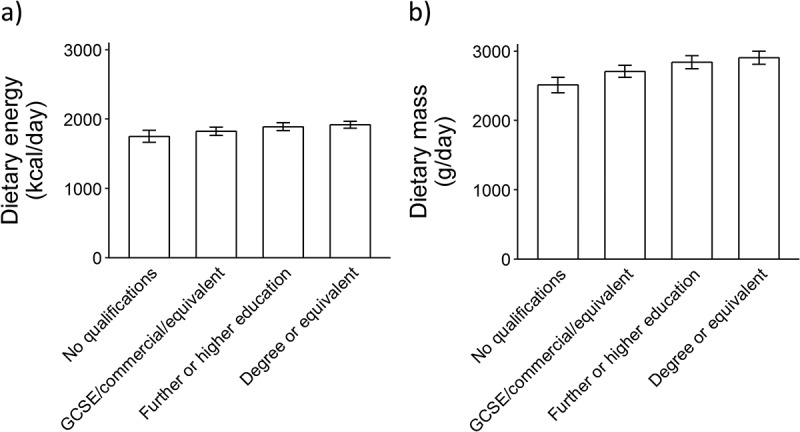
Bar chart indicating the age- and sex-adjusted (a) mean dietary energy intake and (b) mean dietary mass intake and 95% confidence interval by educational attainment using survey representative UK data for UK adults from the National Diet and Nutrition Survey (2008–2012) (*n* = 2083).^16^

Beyond foods, it is important to consider the most appropriate metric with which to compare diets, because the overall diet is ultimately more important in terms of food security and long-term health than individual foods. We suggest that cost per unit of energy is the most appropriate metric for comparing the cost of different diets, given that the total diet should be within a specified level of energy intake based upon age, sex, basal metabolic rate, and physical activity levels, whereas the number of portions or the total mass of a diet can vary depending on the types of foods consumed, the typical portion size, and energy density of individual diets. This can be seen in the limited deviation in dietary energy observed in populations. Figure 1a shows the age- and sex-adjusted means of food energy consumed by adults in the UK by educational attainment based on data from the *National Diet and Nutrition Survey* (2008–2012).^32^ Mean energy intake is tightly clustered across categories of educational attainment, with a maximum difference across groups of 168 kcal/day (10% variation). However, total mass of the diet (Figure 1b) varies substantially and is socially patterned, with higher income groups having a dietary mass that was 392 g/day (16%) higher than that of the lowest income group. The larger variation observed in dietary mass indicates that there is less physiological regulation of how much food mass can be consumed in contrast to energy, which appears to be more consistent across the population. Standardizing dietary costs for levels of energy intake also provides consistency with dietary guidance, in which recommended quantities of foods and nutrients are scaled in relation to energy intake.

In the UK, United States, and other high-income countries, the burden of obesity and some chronic diseases is caused in part by the consumption of diets that contain excess energy but insufficient levels of other nutrients.^33^ ^,^ ^34^ Because sufficient energy consumption alone is not enough to ensure good health in the long term, it would be important to identify ways of improving the nutrient to calorie ratio of the overall diet by consuming more nutrient-rich foods. Such foods might not necessarily be low cost on a per calorie basis but still be economical in terms of the cost per unit of nutrients. Studies of this kind in the United States have combined national food price data with nutrient composition data to identify low-cost, nutritious foods.^31^ ^,35^

Though some have argued that the cost of foods expressed per unit of energy has little behavioral significance,^36^ research indicates that lower income households tend to buy foods that are on average cheaper per calorie, perhaps meaning that there is a behavioral response to low prices for food energy.^37^ ^,^ ^38^ Furthermore, longitudinal data indicate that, irrespective of their socioeconomic position, households shifted to purchasing cheaper calories during a period of rising food prices and falling real incomes.^39^ This indicates that cutting food costs without lowering energy intake can lead to the substitution of foods that provide more energy for a given price, which has been suggested previously in simulation studies.^40^ ^,41^

It is worth noting that our data are from the UK and that we have only considered the arguments in favor of different metrics in the context of a high-income country such as the UK, meaning that they may not necessarily apply to middle- or low-income settings. However, they could reasonably be expected to be appropriate for use in countries with similar economic and agricultural systems, particularly other countries within the European Union that are subject to the same laws governing agricultural subsidy and food production standards. The United Kingdom is a high-income country and despite the price of food rising by 7.7% since 2007, the mean percentage of household expenditure on food is 11.4% and overall UK prices are just 0.5% greater than the European Union average.^42^ ^,43^ Users of our findings should assess whether or not the economic and food system context in which they are working is comparable to that of the UK.

## Conclusions

In this article we have briefly reviewed the debate concerning the most appropriate price metric for the comparison of different foods and beverages, demonstrating the sizeable effect that metric choice can have on the results of price comparisons. In an attempt to outline guidance concerning the use of price comparison metrics, we have made suggestions as to when to apply the different approaches according to the question being addressed. Though there are numerous occasions where it is more appropriate to analyze food prices in terms of mass or portion size, when the research concerns public health and the ability to eat healthily, we argue that energy is often the most appropriate metric for assessing the cost of both foods and diets.
